# Simulation of the contractile response of cells on an array of micro-posts

**DOI:** 10.1098/rsta.2009.0097

**Published:** 2009-09-13

**Authors:** J. P. McGarry, J. Fu, M. T. Yang, C. S. Chen, R. M. McMeeking, A. G. Evans, V. S. Deshpande

**Affiliations:** 1 Department of Mechanical and Biomedical Engineering, National University of Ireland, Galway, Ireland; 2 Department of Bioengineering, University of Pennsylvania, Philadelphia, PA 19104, USA; 3 Mechanical Engineering Department, University of California, Santa Barbara, CA 93106, USA; 4 Materials Department, University of California, Santa Barbara, CA 93106, USA

**Keywords:** mechano-sensitivity, stress fibre, actin, contractility

## Abstract

A bio-chemo-mechanical model has been used to predict the contractile responses of smooth cells on a bed of micro-posts. Predictions obtained for smooth muscle cells reveal that, by converging onto a single set of parameters, the model captures all of the following responses in a self-consistent manner: (i) the scaling of the force exerted by the cells with the number of posts; (ii) actin distributions within the cells, including the rings of actin around the micro-posts; (iii) the curvature of the cell boundaries between the posts; and (iv) the higher post forces towards the cell periphery. Similar correspondences between predictions and measurements have been demonstrated for fibroblasts and mesenchymal stem cells once the maximum stress exerted by the stress fibre bundles has been recalibrated. Consistent with measurements, the model predicts that the forces exerted by the cells will increase with both increasing post stiffness and cell area (or equivalently, post spacing). In conjunction with previous assessments, these findings suggest that this framework represents an important step towards a complete model for the coupled bio-chemo-mechanical responses of cells.

## 1. Introduction

Most living cells sense, support and generate forces central to their functionality (e.g. Harris *et al*. 1981; Bao & Suresh 2003; Discher *et al*. 2005). The relevant forces have been measured using a succession of approaches. The first used continuous polymer substrates to examine the deformations (Harris *et al*. 1981). Later methods improved the resolution by (i) increasing the compliance of the substrates (Burton & Taylor 1997) and (ii) micropatterning islands to facilitate measurements in areas as small as single focal adhesions (Balaban *et al*. 2001). More recently, the distribution of forces exerted by a cell has been measured by seeding cells on a bed of poly(dimethylsiloxane) (PDMS) micro-posts and by determining the independent deflections of the posts (Tan *et al*. 2003). The interpretation of these measurements has been challenging because cells undergo remodelling and reorganize their cytoskeleton in response to their mechanical environment. Most attempts have taken the perspective that the cytoskeleton is an interlinked structure of passive filaments (Satcher & Dewey 1996; Storm *et al*. 2005). Contractility, when included, has been imposed as a stress-free strain on the cell, regarded as either an isotropic elastic continuum (Nelson *et al*. 2005) or a discrete set of elastic filaments (Mohrdieck *et al.* 2005). All such models neglect the biochemistry of the active apparatus within the cell that generates, supports and responds to mechanical forces. To address these deficiencies, Deshpande *et al*. (2006) have introduced a bio-chemo-mechanical model (appendix A) with a demonstrated capability of explaining a wide variety of observations including: (i) the influence of cell shape and boundary conditions on the development of anisotropy with the cytoskeleton (Deshpande *et al*. 2008); (ii) the distributions of actin and vinculin in cells on substrates with patterned fibronectin patches (Pathak *et al*. 2008); and (iii) the formation of stress fibres perpendicular to the direction of cyclic stretching (Wei *et al*. 2008). The objective of this paper is to assess whether the same model can be used to interpret measurements made using cells on a bed of micro-posts.

Micro-post experiments have revealed the following. (i) The steady-state average force per post, 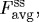 increases with increasing post stiffness (Saez *et al*. 2005). (ii) For 5×5 (or smaller) arrays of posts, 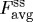 increases with increasing number of posts (Tan *et al*. 2003) but decreases for arrays with 10×10 (or more) posts (Yang *et al*. 2007). (iii) Correlations exist between the forces and stress fibre distributions, established through actin staining and from the curvatures of the cell membrane between posts (Tan *et al*. 2003; Sniadecki *et al.* 2007). For the Deshpande *et al.* (2006) model to be viable, it must be capable of simultaneously predicting *all of these phenomena*. For assessment purposes, the observations of Tan *et al*. (2003) made with smooth muscle cells are used to demonstrate that the force measurements, the actin distributions and the cell membrane curvatures can all be self-consistently interpreted within the framework of the model, upon selecting a specific set of parameters. To assess generality, the same model is used to rationalize observations made on fibroblasts (Yang *et al*. 2007), augmented by some additional measurements on fibroblasts as well as on mesenchymal stem cells (MSCs). Finally, the model is used to make additional predictions of the effects of the stiffness and geometry of the post array on the response of smooth muscle cells.

## 2. Synopsis of the modelling approach

**Figure 1.**
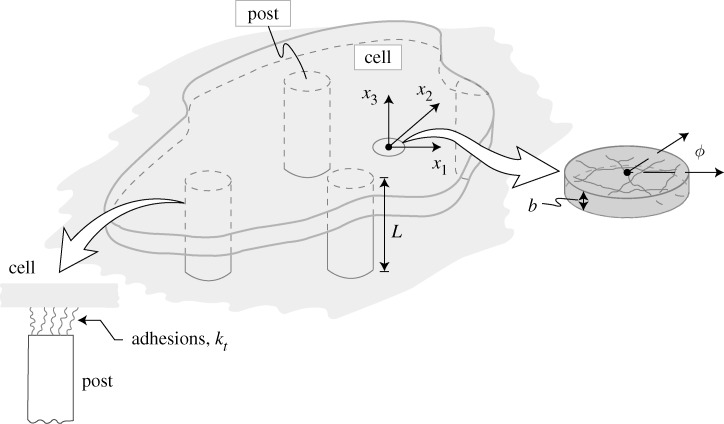
Sketch of a two-dimensional cell lying on a bed of micro-posts. The two insets show the unit cell with the network of stress fibres and the adhesion model of the cell to the posts.

We envisage a two-dimensional cell of thickness *b* lying in the *x*_1_−*x*_2_ plane on a bed of PDMS micro-posts with the PDMS substrate having its normal along the *x*_3_-direction (figure 1). The PDMS posts are elastic, with the cell attached only to the post tops (figure 1). The main aspects of the model are summarized in appendix A, along with the appropriate references. Unless otherwise specified, we restrict our attention to cells that are constrained to spread across square arrays of *N*×*N* posts.

### (a) Correlation between simulation outputs and observations

To reveal the stress fibre distributions, cells are stained by immunofluorescence for the protein actin. Most immunostaining techniques only image the dominant stress fibres. The fine meshwork of filaments is not typically visible when standard epifluorescence or confocal microscopes are used. Consequently, when stress fibres are uniformly distributed in all orientations, a fluorescence micrograph stained for filamentous actin displays a uniform background. By contrast, when the actin concentration is markedly anisotropic, bundling of actin–myosin contractile units in the form of stress fibres is evident in a fluorescence micrograph. Thus, to correlate the observations with the predictions, we define a measure of the circular variance of the stress fibre concentrations as 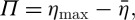, where 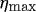 is the maximum polymerization level, which occurs at orientation ϕ_*S*_, while 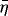 is an average value defined as 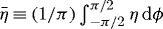; see appendix A for a definition of the stress fibre concentration η(ϕ). The value of Π varies from 0 to 1, corresponding to perfectly uniform and totally aligned distributions, respectively. Subsequently, we show that the distributions of Π predicted by the model consistently correspond with the actin images obtained by immunofluorescence staining.

We also compare measurements and predictions of the forces exerted by the cell on the PDMS micro-posts. Two measures will be employed for comparisons: (i) the cell traction force on the individual posts, with 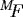 denoting the magnitude of the force on the *M*th post; and (ii) the average force *F*_avg_ on all posts that are attached by the cell, where *F*_avg_ is defined as 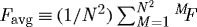.

## 3. Smooth muscle cells

**Figure 2.**
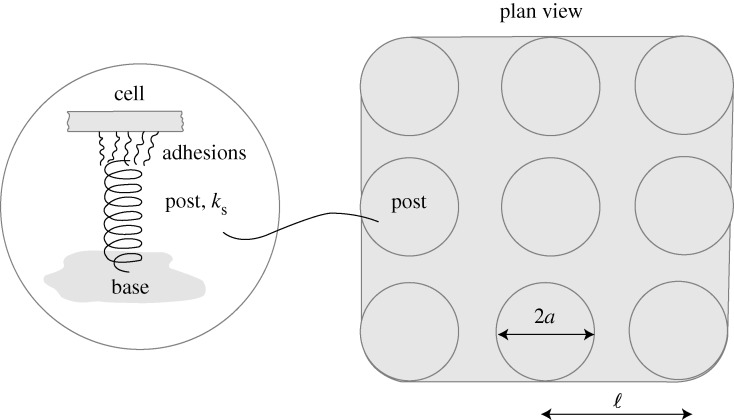
Sketch of the model of a square cell on a bed of 3×3 posts. The cell completely covers all the post tops with which it is in contact. A sketch of the model for the posts comprising a rigid circular disc and springs is also included.

Traction force measurements for smooth muscle cells have been performed on a square array of PDMS micro-posts (figure 2) with a post radius *a*=1.5 μm, bending stiffness *k*_s_=32 nN μm^−1^ and a post centre-to-centre spacing ℓ=10 μm (Tan *et al*. 2003). Cell spreading was controlled in those experiments by using the microcontact printing method to print fibronectin on the tops of selected posts. The cells were serum starved for 12 h and then exposed to lysophosphatidic acid (LPA). We define time *t*=0 (when the signal is applied; equation (A1)) as the instant when the cells are exposed to LPA. At that instant, we assume that the cells have spread over the adherent posts and have square shape, except at the rounded corners, where they cover the posts (figure 2).

**Figure 3.**
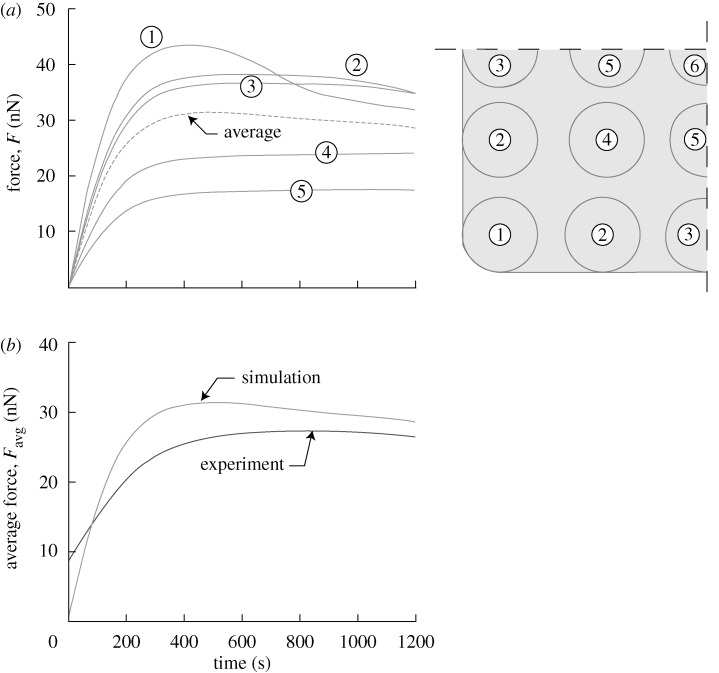
Response of smooth muscle cells on a bed of 5×5 posts investigated in Tan *et al*. (2003). (*a*) Predictions of the deflection versus time histories (a sketch that illustrates the post numbering is also included). (*b*) Comparison between the predicted and measured temporal variations of *F*_avg_. By symmetry, no resultant force is exerted on the central post labelled 6 in (*a*).

**Figure 4.**
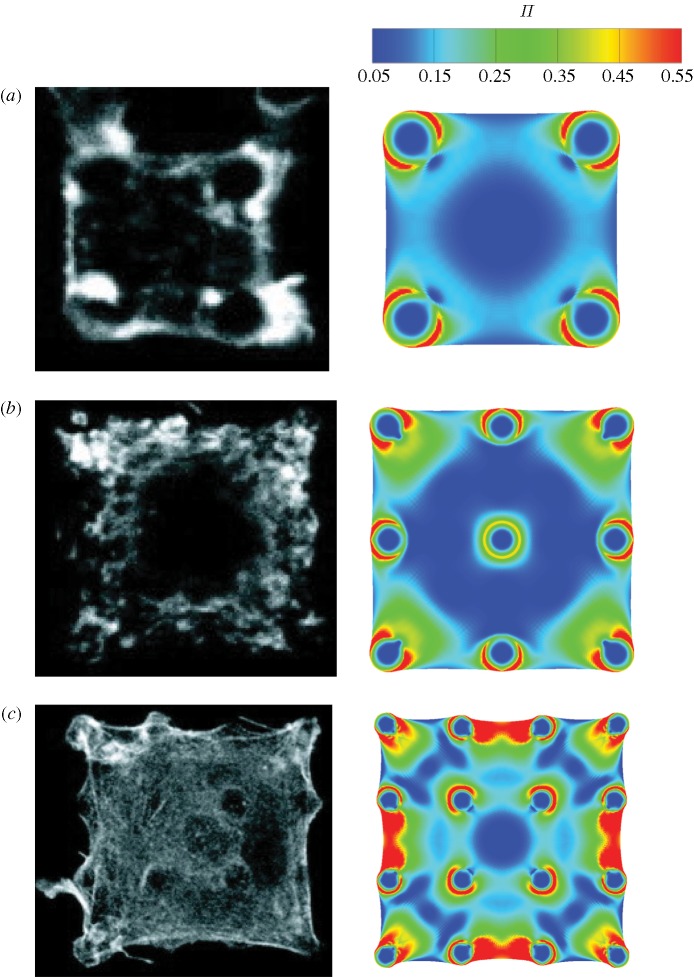
A comparison between the observed and simulated steady-state actin distributions in smooth muscle cells on (*a*) 2×2, (*b*) 3×3 and (*c*) 4×4 arrays of posts. The observations are from Tan *et al*. (2003) and the simulations show actin distributions as parametrized by Π.

### (a) Choice of parameters

A set of parameters for the contractility model has been sought that not only resides in accepted ranges but also gives the best correspondence between measurements and predictions *of all three of the following metrics*: (i) the actin distributions at steady state (attained in about five minutes after LPA stimulation); (ii) the transient and steady-state deflections of the micro-posts; and (iii) the curvature of the cell membrane between the posts. The rate parameters in the model were adjusted to reflect the measured time scale and were set to 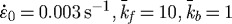 and θ=70 s. The parameters governing the contractility are the Hill constant 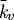 (cf. equation (A 3)) and the maximum tensile stress 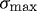 that a stress fibre bundle can exert. It will emerge below that the choices 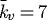 and 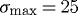 kPa are the only ones that provide acceptable predictions of all of the foregoing measurements. Other choices might give agreement for one metric, but not all three. The passive elastic properties of the cell do not significantly affect the contractile response. We thus assign commonly accepted values of the Young modulus *E* and the Poisson ratio ν for the smooth muscle cells (Boal 2002): *E*=0.4 kPa and ν=0.3. Similarly, the adhesion parameters do not affect the present results (appendix B) so we invoke values consistent with data presented in Lauffenburger & Linderman (1996): stiffness for the focal adhesion complex, *k*_ FA_=0.15 nN μm^−1^ and a ligand–receptor density of ξ=3333 μm^−2^ (corresponding to a focal adhesion stiffness *k*_*t*_=ξ*k*_*FA*_=500 nN μm^−3^). We note that these parameters are not significantly different from those used in previous investigations (e.g. Deshpande *et al*. 2006; Pathak *et al*. 2008) for fibroblasts and retinal endothelial cells, respectively.

**Figure 5.**
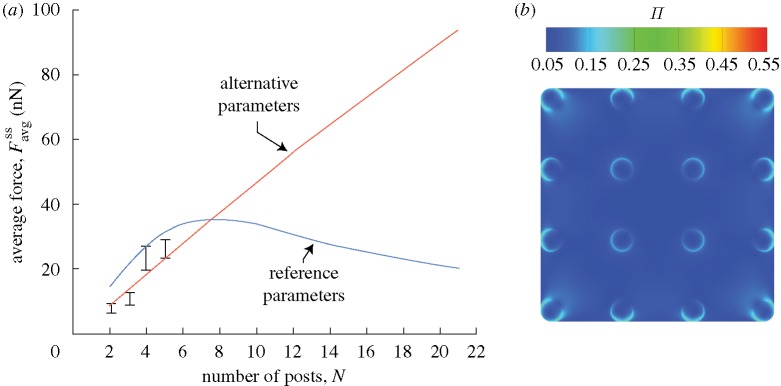
(*a*) The predictions of the variation of the steady-state value of the average force per post, 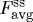, as a function of *N* for smooth muscle cells on an array of *N*×*N* posts. Predictions are shown for two sets of cell parameters labelled ‘reference’ and ‘alternative’. The corresponding measurements from Tan *et al*. (2003) are also included (error bars). (*b*) The corresponding predictions of the steady-state actin distributions (as parametrized by Π) in the cell on 4×4 posts with *alternative properties* (i.e. 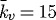 and 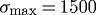 kPa but all other properties fixed at their reference values).

### (b) Comparisons with measurements

#### (i) Time evolution of forces exerted on posts

The predicted time variation of the forces exerted on the posts is plotted in figure 3*a*. The consistency with the measurements is succinctly illustrated in figure 3*b*, where only the average force per post, *F*_avg_, is included.

#### (ii) Actin distributions

Beyond the peak, the forces undergo negligible change, hereafter referred to as ‘steady state’. Comparisons between the measured steady-state actin distributions and corresponding theoretical predictions of Π are included in figure 4 for cells plated on arrays of 2×2, 3×3 and 4×4 posts. The observed features vividly reproduced in the simulations include: (i) concavity of the cell membrane between the posts; (ii) highest actin concentration near the cell perimeter and immediately adjacent to the posts on the cell periphery; and (iii) a central region with a low actin concentration (especially clear for the cells on the 2×2 and 3×3 post arrays). Undoubtedly, there exist some discrepancies between the observations and predictions—notably, the curvature of the cells between the posts is slightly under-predicted by the model. We attribute this to the fact that many elements of the cytoskeleton such as microtubules and intermediate filaments are not explicitly modelled and a more refined analysis is required to increase the fidelity of the predictions.

#### (iii) Scaling of forces with number of posts

**Figure 6.**
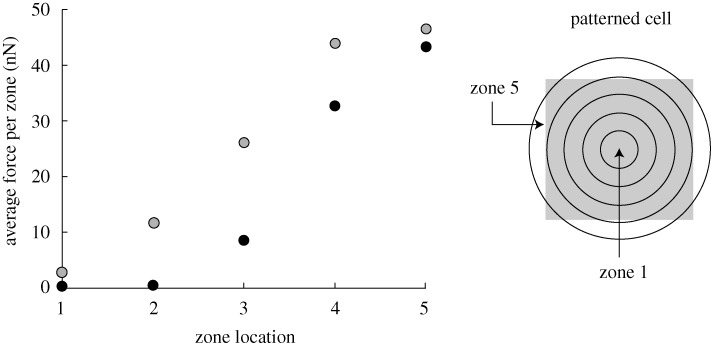
The variation of the average post force with position in smooth muscle cells on 11×11 (grey circles) and 21×21 (black circles) arrays of posts. The post force is averaged over five annular rings of equal width as illustrated.

The steady-state average forces (denoted as 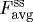 corresponding to the peak value of *F*_avg_) are plotted in figure 5*a* as a function of *N*. The predictions are in excellent agreement with the measurements of Tan *et al*. (2003). Two regimes emerge from the theoretical model: upscaling for *N*<8, where 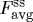 increases with increasing *N*, and downscaling for large *N*, where 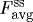 decreases with increasing *N*. These two regimes can be rationalized as follows. When the number of posts that are attached by the cell is small, the contraction of stress fibres located in the cell interior is resisted by *all of the attached posts*, resulting in high levels of stress fibre polymerization as the stress fibres are near the isometric state (see equation (A2)). This causes the traction force exerted by the cells to increase as *N* increases. For a cell seeded on a very large number of micro-posts, the cell interior is under uniform tension, with no resultant traction forces on the posts. Namely, only posts towards the cell periphery experience a resultant traction force, causing the average 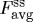 to decrease with increasing *N*. As a corollary, we note that the forces on the peripheral posts are higher than those near the interior. To illustrate this observation further, the average forces exerted on individual posts in five annular zones of equal width are plotted in figure 6 for cells plated on 11×11 and 21×21 post beds: clearly the average traction force exerted on individual posts increases from zone 1 to zone 5.

**Figure 7.**
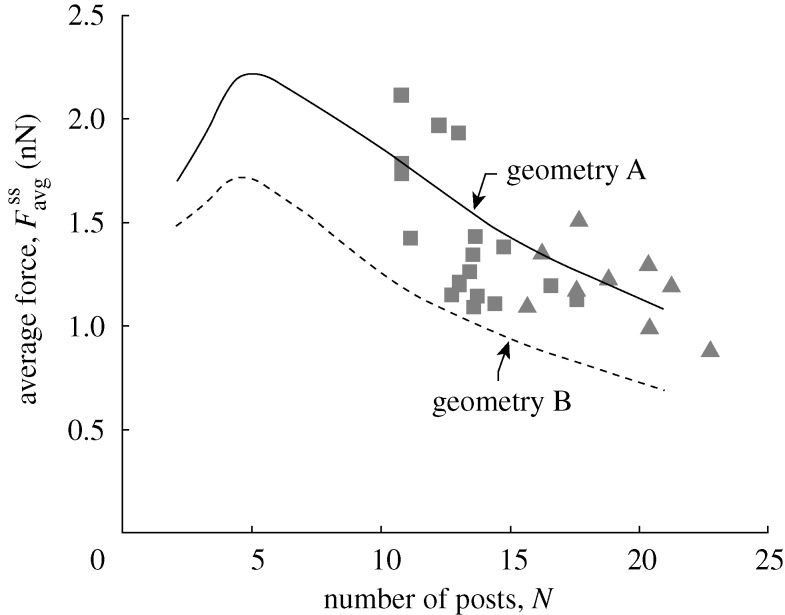
The predictions of the variation of the steady-state value of the average force per post, 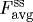, as a function of *N* for fibroblasts on an array of *N*×*N* posts. The corresponding measurements from Yang *et al*. (2007) are also included (squares, geometry A; triangles, geometry B). Data and predictions are shown for two sets of post geometries.

#### (iv) Sensitivity to parameter choice

Predictions of the variation of 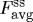 with *N* are included in figure 5*a* for the choice of parameters 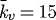 and 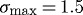 MPa (marked as ‘alternative’ in figure 5*a*), with all other properties fixed at their reference values. This rather different set of parameters also predicts values of 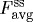
consistent with the experimental measurements obtained by Tan *et al*. (2003). However, there are two key differences. (i) The relationship between the average force and the number of posts is linear and no downscaling regime emerges for *N*<20. (ii) Moreover, the levels of Π are low and there is almost no curving of the cell membrane between the posts; see figure 5*b* for an example of a cell seeded on 4×4 posts. This prediction is clearly contrary to experimental observations (figure 4*c*). A wide-ranging numerical study revealed that only the reference set of parameters gave good correspondence for all three metrics.

In summary, by converging onto a single set of the parameters, it has been possible to use the contractility model to predict all of the responses observed for smooth muscle cells in a self-consistent manner.

## 4. Fibroblasts

Fibroblasts exert considerably smaller contractile forces than smooth muscle cells. Consequently, to implement the model, 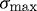 must be recalibrated (all other parameters remain fixed). For this purpose, the contractility measurements conducted with NIH/3T3 fibroblasts (Yang *et al*. 2007) on large arrays of nano-posts are used. These measurements have been performed for the following two arrays of posts:

- centre-to-centre post spacing ℓ=4 μm, post radius *a*=0.4 μm and post bending stiffness *k*_s_=16 nN μm^−1^; and
- centre-to-centre post spacing ℓ=2.5 μm, post radius *a*=0.4 μm and post bending stiffness *k*_s_=28 nN μm^−1^.

In this arrangement, the cells spread over 200 to 600 posts, but their shape varied between each measurement. Since the cell shapes were not reported, we assume square cells spread over the appropriate number of posts. Good correspondence between measurements and predictions is obtained by decreasing 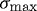 from 25 kPa for smooth muscle cells to 3.25 kPa for fibroblasts (figure 7). Note that, in contrast to smooth muscle cells, 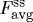
*decreases with increasing N* for *N*>8. The simulations predict these opposing trends. They also predict that, had the fibroblast cells been restricted to fewer than 5×5 posts, they would also have exhibited an upscaling trend, where average traction force 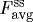 increases with increasing *N*. This theoretical prediction remains to be proved from traction force measurements for NIH/3T3 fibroblasts.

### (a) Actin measurements

**Figure 8.**
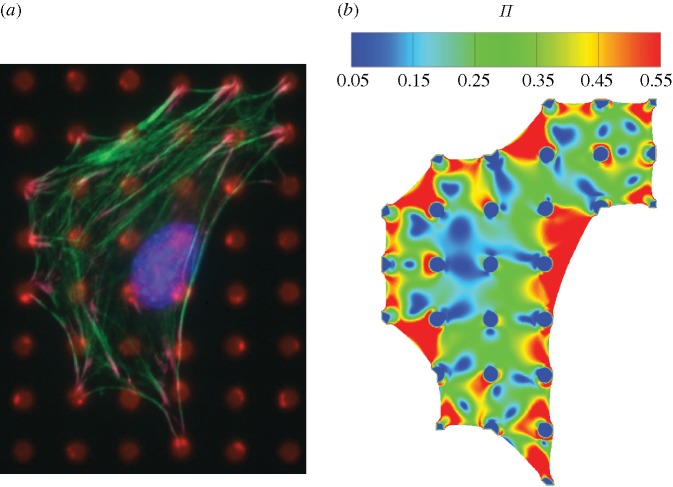
(*a*) Observed steady-state actin distributions in a fibroblast cell spread over 29 posts. (*b*) The corresponding simulation of steady-state actin distribution as parametrized by Π.

Additional measurements have been made of the actin distributions in NIH/3T3 fibroblasts (see Tan *et al*. (2003) for details of the method). These experiments were conducted on the same cell line as Yang *et al*. (2007), but with the cells seeded onto post arrays identical to those employed by Tan *et al*. (2003): namely, a post bending stiffness *k*_s_=32 nN μm^−1^, spacing ℓ=10 μm and radius *a*=1.5 μm. A representative image of a cell stained for actin (green) is included in figure 8*a* (with the nucleus stained in blue). The contractile response has been simulated by assuming an initial cell with straight edges between posts and using the foregoing parameters for fibroblasts. The steady-state predictions of Π (included in figure 8*b*) correspond closely to the experimental observations. In addition, the difference between the measured and predicted values for 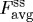 (7.32 and 5.5 nN, respectively) is within the uncertainty of the experimental measurements (the vectorial sum of the measured 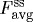 suggests an uncertainty of 1–2 nN).

In summary, once 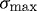 has been recalibrated for fibroblasts, the model satisfactorily captures the trends in the traction forces and in the actin distributions.

**Figure 9.**
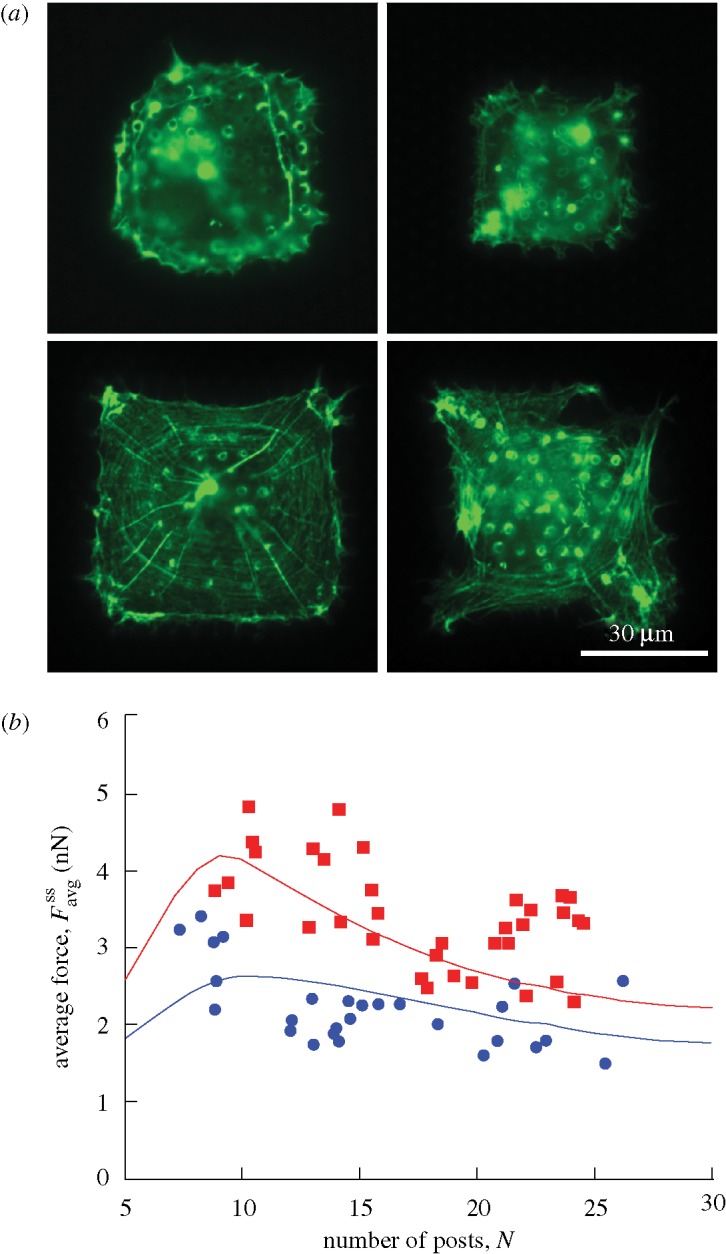
(*a*) Observed steady-state actin distributions in a selection of MSCs. (*b*) Measurements (symbols) and predictions (full curves) of the variation of the steady-state value of the average force per post, 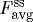, as a function of *N* for MSCs on an array of *N*×*N* posts. Results are shown for two selected values of the post stiffness *k*_*s*_ (*k*_*s*_=7 nN μm^−1^, blue circles; 14 nN μm^−1^, red squares).

## 5. Mesenchymal stem cells

In order to illustrate the generality of the bio-chemo-mechanical model, some additional measurements of the contractility of MSCs were conducted. The experimental procedure followed that detailed in Tan *et al*. (2003) and Yang *et al*. (2007). For these measurements the PDMS posts have a radius *a*=0.9 μm and post centre-to-centre spacing ℓ=4 μm. The post height was varied to give post stiffnesses in the range 7 nN μm^−1^≤*k*_s_≤35 nN μm^−1^.

The following similarities are observed between MSCs, smooth muscle cells and fibroblasts:

- Images of the MSCs stained for actin (green) presented in figure 9*a* reveal that, similar to the smooth muscle cells (figure 4), rings of actin are present around the micro-posts.
- The scaling of the average traction force per micro-post, 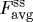, with the number of posts (figure 9*b*) over the range of *N* investigated is similar to that for fibroblasts (figure 7), with 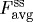 decreasing as *N* increases.

**Figure 10.**
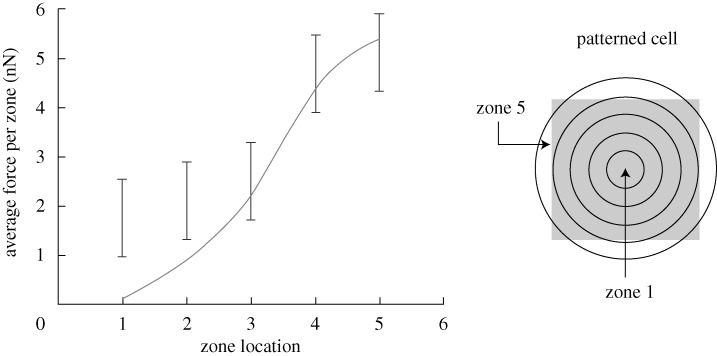
Measured (error bars) and predicted (full curve) values of the variation of the average post force with position in MSCs on an 11×11 array of posts with stiffness *k*_s_=35 nN μm^−1^. The post force is averaged over five annular rings of equal width, as illustrated.

Two additional observations are crucial for MSCs:

- For a given value of *N*, 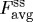 decreases with decreasing post stiffness *k*_s_ (figure 9*b*).
- The average post force measured in five annular rings of equal width (for a square cell on an 11×11 array of posts of stiffness *k*_s_=35 nN μm^−1^) is higher towards the cell periphery (figure 10), consistent with figure 6.

In order to model these measurements, the cell contractility model was re-calibrated for MSCs by decreasing 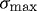 to 8 kPa and employing a Hill constant 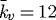 while keeping all other parameters fixed. Good correspondence between measurements and predictions is obtained (within measurement uncertainty) for both the scaling of 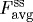 with *N* (figure 9*b*) and the average traction force per post as a function of post position (figure 10). It is worth noting here that the threshold number of posts, when the behaviour of the cells switches from an upscaling to a downscaling response, appears to be dependent on cell type; compare figures 5*a*,  7 and  9*b*. However, recall that Deshpande *et al*. (2007) had shown that this threshold number of posts increases with decreasing post spacing. We thus believe that the primary reason for differences in the threshold values seen between figures 5*a*,  7 and  9*b* is not cell type but rather is related to the fact that the post spacing varied for the different cell types investigated here.

In summary, the contractile response of MSCs on an array of micro-posts exhibits the same qualitative trends as smooth muscle cells and fibroblasts. Further, the contractility model once suitably recalibrated again captures the observations with acceptable accuracy.

## 6. Sensitivity to the properties of the micro-posts

The bio-chemo-mechanical model here is further used to predict the sensitivity of the response of smooth muscle cells to the geometric and mechanical properties of the PDMS micro-posts. All simulations are presented for initially square-shaped cells (*b*=1 μm) on a cubic array of *N*×*N* posts. The post array properties to be independently varied are the post spacing ℓ, radius *a* and bending stiffness *k*_s_ (*k*_s_ can be varied independently of *a* by adjusting the post height *L* or the choice of material). The simulation results are presented in terms of the non-dimensional stiffness, 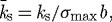 and geometric variables of the array,6.1
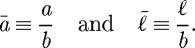

The non-dimensional steady-state average traction force per post is then defined as6.2
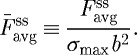



### (a) Effect of post stiffness

**Figure 11.**
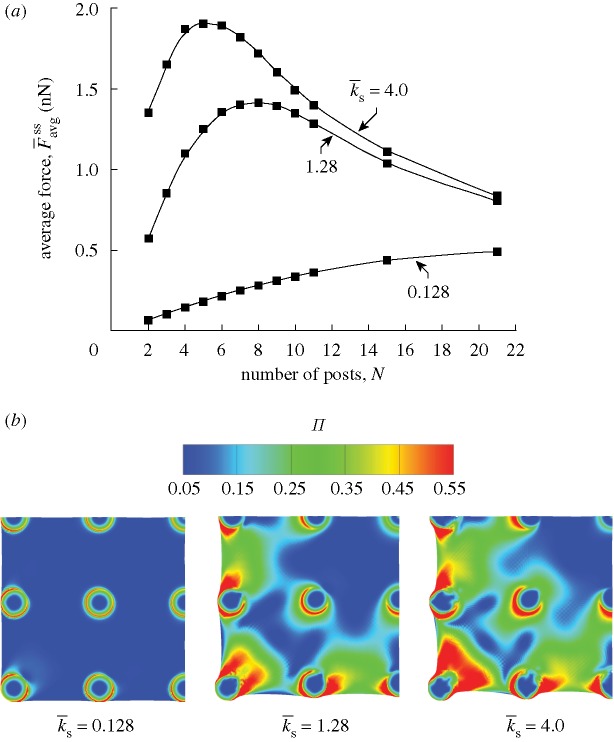
Simulations for muscle cells on a bed of *N*×*N* posts with 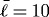 and 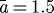. (*a*) Predictions of the variation of the normalized steady-state force averaged over all posts, 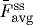, with *N* for three choices of the non-dimensional post stiffness 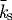. (*b*) The corresponding simulations of steady-state actin distribution as parametrized by Π for cells on a bed of 5×5 posts. Results are shown for the 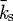 values in (*a*) and, for the sake of clarity, only a quarter of the cells are shown.

Simulation results for three choices of micro-post stiffnesses (figure 11*a*) affirm that 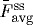 first increases with *N*, reaches a maximum and then decreases. For small numbers of attached posts *N*, the force 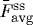 markedly increases with increasing 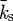. This observation is rationalized as follows. The contraction of the cells on stiff posts is more constrained (i.e. cells remain closer to their isometric state), resulting in higher levels of actin polymerization and consequently higher contractile forces (cf. equation (A2)). Moreover, similar to the observations in figure 9*b*, 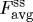 increases with increasing 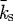 for a given number of posts *N*. The predicted increase in actin concentration with increasing 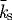 is further illustrated in figure 11*b*; figure 11*b* also indicates that the higher contractility of cells on stiff posts results in significant curving of the cell membrane between the posts. These results are consistent with numerous experimental findings (e.g. Saez *et al*. 2005), but seem to be at odds with the observations of Freyman *et al*. (2002), which suggested that the forces exerted by fibroblasts are independent of substrate stiffness. Recall that in the ‘cell monitor’ of Freyman *et al*. (2002), cells are motile and permitted to spread in the collagen matrix. This situation is different from the stationary cells investigated here and is the most likely cause for the apparent discrepancy.

### (b) Effect of array geometry

**Figure 12.**
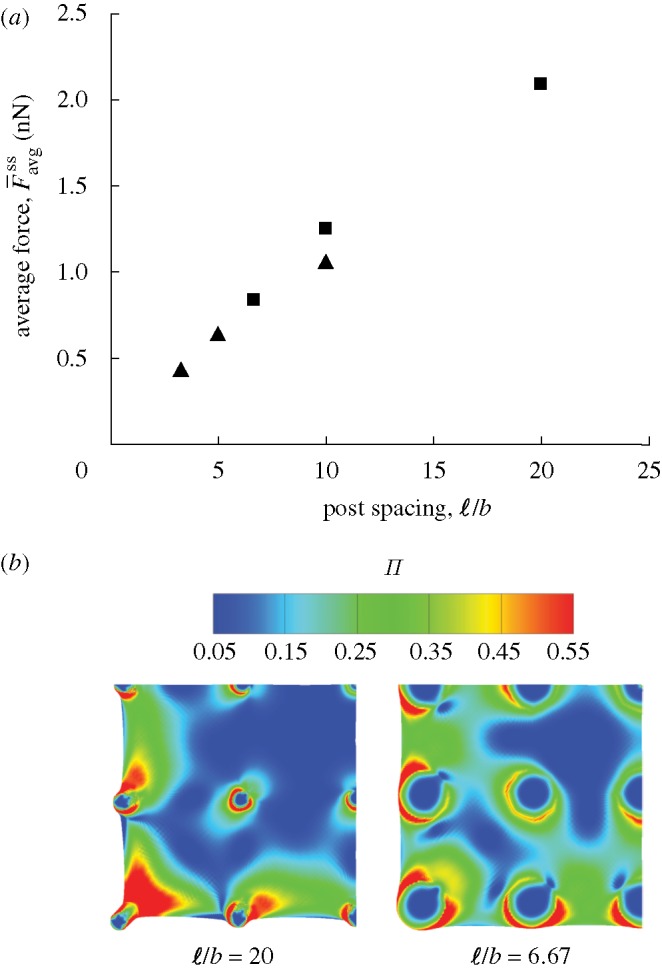
Simulations for muscle cells on a bed of 5×5 posts with stiffness 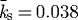. (*a*) Predictions of the variation of the normalized steady-state force averaged over all posts, 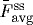, with normalized post spacing 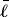 for two choices of the non-dimensional post radius 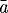 (filled square, *a*/*b*=1.5; filled triangle, *a*/*b*=0.75). (*b*) The corresponding simulations of steady-state actin distribution as parametrized by Π for cells with 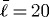 and 6.67 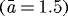. For the sake of clarity, only a quarter of the cells are shown.

The predicted 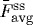 are plotted in figure 12*a* as a function of 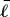 for two selected values of 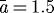 and 0.75. While doubling 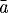 only marginally increases 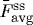, increasing 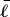 has a substantial effect on 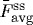, suggesting that the traction forces increase with increasing cell area. This influence of post spacing on traction force is explained as follows. For a given cell contractile force, the post deflections remain fixed, but the strain in the cell decreases with increasing ℓ. Thus, for larger ℓ, the strain rates within the cell are smaller (the cell is closer to its isometric state) and hence cells achieve higher levels of actin polymerization, resulting in higher traction forces. The predicted steady-state distributions of Π are included in figure 12*b* for 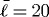 and 6.67 (cells on the 5×5 post arrays with 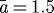). In line with these arguments, Π and the curvature of the cell boundaries are significantly higher for the larger cell 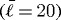.

## 7. Concluding remarks

A bio-chemo-mechanical model has been used to predict the contractile responses of cells on a bed of micro-posts. By manipulating two parameters, the Hill constant 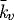 and the maximum tensile stress 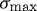 exerted by a stress fibre bundle, the bio-chemo-mechanical model is able to predict the contractile response of three different cell types on a bed of PDMS micro-posts. It is not surprising that these cell properties vary with cell type owing to the expression of different isoforms of actin and myosin. There are at least six isoforms of actin expressed in mammalian cells. Non-muscle cells such as fibroblasts express beta and gamma actin (Herman 1993) while alpha actin is predominant in all types of muscle cells (Kashina 2006). In addition to expressing different isoforms of actin, different cell types may express different isoforms of myosin II. The differential expression of actin and myosin II isoforms in different cell types can affect protein distribution and kinetics, which may explain the need to use different modelling parameters in predicting the contractile responses in cells.

We have demonstrated that, by converging onto a single set of parameters, the contractility model predicts all of the responses observed for smooth muscle cells in a self-consistent manner. The effects successfully predicted include: (i) the scaling of the force exerted by the cells with the number of posts; (ii) actin distributions within the cells, including the rings of actin around the micro-posts; (iii) the curvature of the cell boundaries between the posts; and (iv) the higher post forces towards the cell periphery. Similar correspondences between predictions and measurements have been demonstrated for fibroblasts and MSCs once the maximum stress exerted by the fibre bundles and the Hill constant have been recalibrated. We emphasize that this is not merely a fitting exercise, as only a consistent set of parameters gives agreement between observations and predictions of three independent sets of metrics, *viz*. the forces exerted by the cells, the actin distributions and the deformed cell shapes. Consistent with observations, it has been shown that the model predicts that the forces exerted by the cells will increase with both increasing post stiffness and cell area (or equivalently post spacing). Previously, it has been shown that the model successfully predicts a diversity of other observations (Deshpande *et al*. 2006, 2007; Pathak *et al*. 2008; Wei *et al*. 2008). Taken together, these findings suggest that the current framework represents a step towards developing a complete model for the coupled bio-chemo-mechanical responses of single cells.

## Figures and Tables

**Figure 13.**
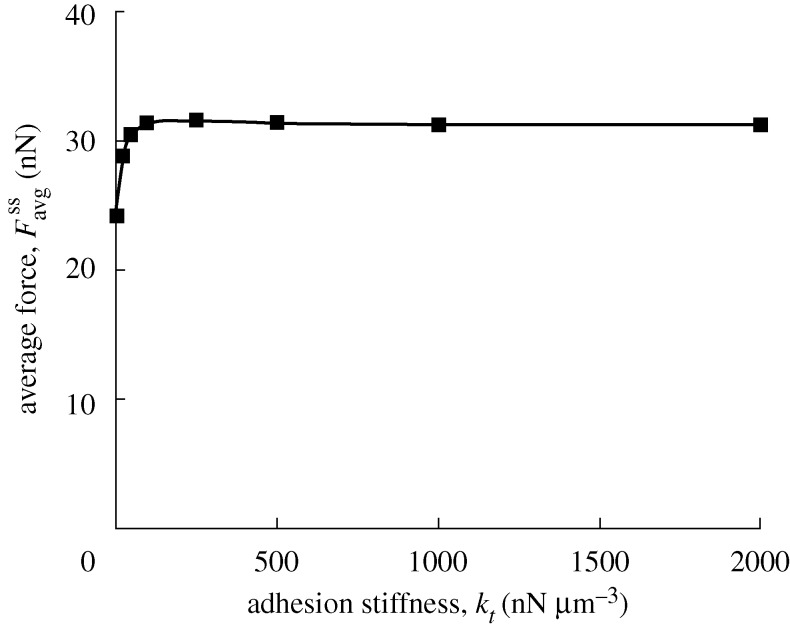
Predictions of the variation of the steady-state force averaged over all posts, 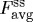, with the adhesion stiffness *k*_*t*_. Results are shown here for the smooth muscle cells on a bed of 5×5 posts as employed in the investigation of Tan *et al*. (2003).

## Appendix A. Cell contractility model

### (a) Stress fibre contractility

A bio-chemo-mechanical model has been devised that captures the formation and dissociation of stress fibres, as well as the associated generation of tension and contractility (Deshpande *et al*. 2006, 2007). The stress fibre formation is initiated by a nervous impulse or a biochemical or mechanical perturbation that triggers a signalling cascade within the cell. We model this signal as an exponentially decaying pulse having level *C* (which may be thought of as the concentration of Ca^2+^) given by

A1

where θ is the decay constant and *t* is the time after the onset of the most recent activation signal. The formation of stress fibres is parametrized by an activation level, designated η (0≤η≤1), defined as the ratio of the concentration of the polymerized actin and phosphorylated myosin within a stress fibre bundle to the maximum concentrations permitted by the biochemistry. The evolution of the stress fibres at an angle ϕ with respect to the *x*_1_-axis (figure 1) is characterized by a first-order kinetic equation,

A2

where the overdot denotes time differentiation. In this formula, σ(ϕ) is the tension in the fibre bundle at orientation ϕ, while  is the corresponding isometric stress at activation level η. Here,  is the tensile stress at full activation (η=1). The dimensionless constants  and  govern the rates of stress fibre formation and dissociation, respectively. In turn, the stress σ is related to the fibre contraction/extension rate  by the cross-bridge cycling between the actin and myosin filaments. The simplified (but adequate) version of the Hill-like equation (Hill 1938) employed to model these dynamics is specified as

A3

where the rate sensitivity coefficient, , is the fractional reduction in fibre stress upon increasing the shortening rate by . We note that, using this Hill-like prescription for the stress versus strain rate relation, the stress fibres act as cables that can sustain tensile stresses but not compressive stresses. This is consistent with the recent work of Bischofs *et al*. (2008) where they argue that cable-like, rather than spring-like, elements are required to give accurate predictions of cell deformation.

A two-dimensional constitutive description for the stress fibre assembly has been derived by noting that the axial fibre strain rate  at angle ϕ is related to the material strain rate  by

A4

The average stress generated by the fibres follows from a homogenization analysis as

A5

The constitutive description for the cell is completed by including contributions from passive elasticity, attributed to intermediate filaments and microtubules of the cytoskeleton attached to the nuclear and plasma membranes. These act in parallel with the active elements, whereupon additive decomposition gives the total stress:

A6

Here, δ_*i**j*_ is the Kronecker delta (for a linear response), *E* the Young modulus and ν the Poisson ratio. These equations are valid in a small or infinitesimal deformation setting; readers are referred to Deshpande *et al*. (2007) for the finite strain and three-dimensional generalization.

### (b) Cell adhesion

The focus of this study is on the contractile response of cells on a bed of micro-posts. Thus, we employ a simplified model for the adhesion between the two-dimensional cell and the flat tops of the micro-posts so as to simplify the interpretation of the results. The shear traction *T*_*i*_ between the cell membrane in contact with the post and the flat top of the post is assumed to be given by a linear elastic relation,

A7

Here *k*_*t*_ is the shear stiffness of the adhesion and Δ_*i*_ the relative sliding displacement between the cell membrane and post. This simplified adhesion model assumes no cooperativity between the stress fibre contractility and focal adhesion formation and growth; readers are referred to Deshpande *et al*. (2008) and Pathak *et al*. (2008) for a comprehensive analysis of cell adhesion. Rather, equation (A7) can be interpreted in terms of focal adhesions as follows. If the number of integrin molecules participating in the adhesions is assumed to be constant, the stiffness is *k*_*t*_=ξ*k*_FA_, where ξ is the concentration of focal adhesion complexes per unit cell membrane area, and *k*_FA_ the associated stiffness of the focal adhesion complex. We demonstrate in appendix B that the results presented here are insensitive to the choice of the parameter *k*_*t*_ to within a realistic range of focal adhesion stiffnesses and concentrations.

### (c) Micro-posts

The micro-posts are vertical cantilevers that deflect in response to the forces exerted by the cells. In the experiments considered here, the elastic micro-posts undergo small deflections, with the force *F* applied to the micro-post related to the deflection δ via the relation

A8

where *E*_*n*_ is the Young modulus of the post material (PDMS in this case) and *I* and *L* the second moment of area of the post cross-section and the post height, respectively. The rotation θ of the ends of the cantilever with respect to the *x*_3_-axis (figure 1) is related to δ as

A9

Thus, for deflections δ that are small compared with the length *L* of the cantilever, it suffices to consider only the deflections of the micro-posts and neglect their rotation. We thus model the posts as rigid circular discs constrained to move in the *x*_1_−*x*_2_ plane (figure 1). The displacement *d*_*i*_ of the discs within the *x*_1_−*x*_2_ plane is constrained by a spring of stiffness *k*_s_ (as sketched in figure 2) such that the force *F*_*i*_ applied by the cell is related to *d*_*i*_ via the relation

A10

The radius *a* of the discs, the micro-post spacing ℓ and the geometrical arrangement of the micro-posts are chosen to match those employed in the experiments.

### (d) Finite element implementation

The model for the cytoskeletal contractility has been implemented as a user-defined material (UMAT) in the commercial finite element (FE) code Abaqus^1^ while the adhesion between the cell and the flat discs representing the tops of the micro-posts was included in the model by employing the user-defined interface (UINTER) option in ABAQUS. In ABAQUS surface interaction terminology we treat the disc representing the micro-post as the *master* surface, whereas the *slave* surface is the entirety of the cell surface. Thus, adhesions are allowed to form only where the cell is in contact with the discs: a portion of the cell surface initially in contact with the disc can lose contact as the cell contracts; in such cases the adhesion between that portion of the cell surface and the micro-post is assumed to break. The linear springs constraining the displacement of the discs representing the post tops are modelled using the SPRING-A option in ABAQUS.

The cells were modelled using deformable four-noded membrane elements (M3D4 in the Abaqus terminology) and the response solved for in a finite strain setting (i.e. the effects of geometry changes on the momentum balance and rigid body rotations are taken into account). Typical FE meshes comprised elements of average size 0.25 μm (for cells with a leading dimension of ≈50 μm) with the meshes refined near the post peripheries to capture large parameter gradients. We conducted mesh sensitivity studies and confirmed that reducing the mesh size below 0.25 μm did not change the results appreciably.

## Appendix B. Effect of the focal adhesion stiffness

In all calculations, the stiffness of the adhesion between the cell and the posts is *k*_*t*_≡ξ*k*_FA_=500 nN μm^−3^. This follows from an assumed ligand–receptor density of ξ=3333 μm^−2^ over the cell membrane and a focal adhesion complex stiffness *k*_FA_=0.15 nN μm^−1^. Over a biologically realistic range in the values of *k*_FA_ and ξ, this choice of stiffness has a negligible effect on the response of cells. Consider a smooth muscle cell on a bed of 5×5 posts (stiffness *k*_s_=32 nN μm^−1^) analysed in §3. The predicted force  (plotted in figure 13 as a function of *k*_*t*_) is approximately constant for *k*_*t*_≥20 nN μm^−1^, but decreases sharply at lower *k*_*t*_ when the stiffness of the adhesions becomes sufficiently low that the cell ‘slips off’ the posts. Based on estimates for *k*_FA_ and ξ given in Lauffenburger & Linderman (1996), we do not anticipate *k*_*t*_ to drop below 100 nN μm^−1^, and thus the results presented here are not sensitive to the precise choice of *k*_*t*_.

A similar study with the fibroblast parameters confirmed that the choice of *k*_*t*_=500 nN μm^−3^ is within the regime where the response is insensitive to the value of *k*_*t*_.
